# Testing the Validity and Reliability of the Shame Questionnaire among Sexually Abused Girls in Zambia

**DOI:** 10.1371/journal.pone.0123820

**Published:** 2015-04-16

**Authors:** Lynn T. M. Michalopoulos, Laura K. Murray, Jeremy C. Kane, Stephanie Skavenski van Wyk, Elwyn Chomba, Judith Cohen, Mwiya Imasiku, Katherine Semrau, Jay Unick, Paul A. Bolton

**Affiliations:** 1 Social Intervention Group, Global Health Research Center of Central Asia, School of Social Work, Columbia University, New York, New York, United States of America; 2 Center for Refugee and Disaster Response, Department of International Health, Bloomberg School of Public Health, Johns Hopkins University, Baltimore, Maryland, United States of America; 3 Department of Mental Health, Bloomberg School of Public Health, Johns Hopkins University, Baltimore, Maryland, United States of America; 4 Ministry of Community Development, Mother Child Health (MCDMCH), Lusaka, Zambia; 5 Center for Traumatic Stress in Children & Adolescents, Allegheny General Hospital, Drexel University College of Medicine, Pittsburgh, Pennsylvania, United States of America; 6 School of Medicine, University of Zambia, Lusaka, Zambia; 7 Center for Global Health & Development, Boston University School of Public Health, Boston, Massachusetts, United States of America; 8 School of Social Work, University of Maryland, Baltimore, Maryland, United States of America; Örebro University, SWEDEN

## Abstract

**Purpose:**

The aim of the current study is to test the validity and reliability of the Shame Questionnaire among traumatized girls in Lusaka, Zambia.

**Methods:**

The Shame Questionnaire was validated through both classical test and item response theory methods. Internal reliability, criterion validity and construct validity were examined among a sample of 325 female children living in Zambia. Sub-analyses were conducted to examine differences in construct validity among girls who reported sexual abuse and girls who did not.

**Results:**

All girls in the sample were sexually abused, but only 61.5% endorsed or reported that sexual abuse had occurred. Internal consistency was very good among the sample with alpha = .87. Criterion validity was demonstrated through a significant difference of mean Shame Questionnaire scores between girls who experienced 0–1 trauma events and more than one traumatic event, with higher mean Shame Questionnaire scores among girls who had more than one traumatic event (*p* = .004 for 0–1 compared to 2 and 3 events and *p* = .016 for 0–1 compared to 4+ events). Girls who reported a history of witnessing or experiencing physical abuse had a significantly higher mean Shame Questionnaire score than girls who did not report a history of witnessing or experiencing physical abuse (*p*<.0001). There was no significant difference in mean Shame Questionnaire score between girls who reported a sexual abuse history and girls who did not. Exploratory factor analysis indicated a two-factor model of the Shame Questionnaire, with an *experience of shame* dimension and an *active outcomes of shame* dimension. Item response theory analysis indicated adequate overall item fit. Results also indicate potential differences in construct validity between girls who did and did not endorse sexual abuse.

**Conclusions:**

This study suggests the general utility of the Shame Questionnaire among Zambian girls and demonstrates the need for more psychometric studies in low and middle income countries.

## Introduction

Child sexual abuse (CSA) is a significant global health problem [1,2]. In a meta-analysis of international samples, mostly from Western high income countries, Pereda and colleagues [3] found that 7.9% of men and 19.7% of women have experienced sexual abuse before the age of 18. In response to CSA, a number of both short and long term outcomes have been indicated, such as substance abuse, risky sexual behavior, anxiety, depression, posttraumatic stress disorder, sleep disorders, and suicide attempts [4,5]. Although the findings are primarily limited to Western cultures, literature has also shown that children frequently experience feelings of shame in reaction to sexual abuse in particular, as the trauma often results in the child developing an intense negative evaluation not just related to their behavior, but to their overall sense of self [6,7]. Subsequently, shame leads to the desire to hide and interrupts the ability to actively cope [6,8]. Girls in particular appear to be at an increased risk of experiencing shame following sexual abuse [6,9]. Some literature has indicated this may be due in part to girls having a greater tendency to self-blame following adverse events [10,11]. Shame has also been found to be a predictor of depression among girls [9]. Finally, as opposed to physical abuse, literature has indicated that sexual abuse is consistently associated with the construct of shame [6,7,12], perhaps due to the typical level of secrecy associated with sexual abuse and violation of personal boundaries associated with it.

Shame has been noted as an important outcome to include in the development of treatments for mental health problems in Western settings [13,14] as it predicts higher levels of dissociation [7], the development and maintenance of PTSD [8,15,16], fear or avoidant behavior with intimate relationships [17,18] and may help distinguish between degree of adjustment among children following CSA [13]. Further, degree of shame has been associated with HIV risk behavior, medication non-adherence and health-related quality of life among individuals living with HIV and a history of CSA [16,19,20]. Chronic experience of shame has also been linked to changes in cortisol levels and related to negative physiological health outcomes [21]. Given these findings, shame is clearly a construct to be measured and targeted as a relevant outcome in the development of evidence-based trauma treatments.

Although adverse outcomes of CSA have been established among Western populations, little attention has been placed specifically on outcomes of CSA in sub-Saharan Africa [22]. In Zimbabwe, Meursing and colleagues [23] anecdotally examined psychological outcomes through in-depth interviews among children who experienced CSA. Results indicated children experienced fear, depression, anger, guilt and behavioral problems [23]. Other research has emphasized risk factors and prevalence estimates of CSA in Sub-Saharan Africa [22,24], but has been limited to studies in South Africa. Further, among the limited research in sub-Saharan Africa on negative outcomes of CSA, we have not found any literature which examines shame.

In order to understand the impact of sexual violence on psychosocial functioning and measure change of symptoms after implementation of programming and/or interventions among CSA survivors in low and middle income countries (LMIC) and specifically in sub-Saharan Africa, appropriate validated measures must be utilized [25]. However, most validated measures in LMIC focus on PTSD and depression among adult trauma-affected populations [e.g., [26,27]. Few validation studies focus on trauma outcomes among children in LMIC and these studies have a similar focus on PTSD [e.g., [28–31]. Through a search of the literature (limited to the English language), we did not find any studies in LMIC that have validated a measure of shame among children who are CSA survivors or have experienced multiple traumatic events.

Similarly, we did not find any studies that have examined the validity of shame utilizing both classical test theory (CTT) and item response theory (IRT) measurement methods. IRT and CTT are two complementary approaches to examining the validity of a latent construct. Item response theory is model-based and accounts for both the person and item location through estimates on the same latent construct [32,33]. The model examines the usefulness of each individual item, which is based on the item’s ability to differentiate among individuals at different points along a latent continuum, in our study representing a range from low to high amounts of shame [32]. Whereas CTT is based on one reliability estimate and one standard error of measurement for the latent trait (based on the total scale score) regardless of the individual’s amount of shame or difficulty of individual items (how much shame is needed to endorse each item), IRT assesses the specific items and specific levels of shame [33,34]. IRT is centered on the relationship between the likelihood that a person will endorse an item in a particular way and the amount of the latent trait being measured that the person has [35,36].

The purpose of this study is to address a gap in the literature by using both IRT and CTT to validate a shame measure used in Zambia among female child survivors of CSA. We aim to outline the process and results of adapting and validating an existing measure of shame among children in Zambia: the Shame Questionnaire [37]. In addition, to specifically assess criterion validity, comparisons are made with the Post Traumatic Stress Disorder-Reaction Index (PTSD-RI) [38], which was previously validated with this population [30].

## Methods

### Background of Study

The current validation study is part of a larger project within Lusaka, Zambia examining the feasibility of implementing evidence-based assessments and treatments for HIV-affected children and adolescents who experienced traumatic events. HIV-affected refers to individuals who are at high risk for HIV infection and/or those who are disproportionately burdened by HIV/AIDS. A qualitative study was conducted in 2004 to increase understanding of problems experienced by HIV-affected women and children in Zambia [39]. Results indicated a high incidence of traumatic events experienced among children and adolescence such as sexual abuse, physical abuse, and domestic violence. At this time, there were no locally validated measures to assess these problems in Zambia.

### Settings and Interviewers

This study was conducted by a record review at the University Teaching Hospital (UTH) in Lusaka, Zambia within a “One-Stop Centre” that provided services for sexually abused youth [40]. The abuse ranged from being sexually touched to intercourse [40]. The purpose of the One Stop Centre was to provide post-exposure prophylaxis for children who were recently sexually abused. Children were brought to the One Stop Centre if someone reported the abuse to a victim support unit or registered the child at the UTH after the abuse occurred. An author of the current study (LKM) trained staff (3 nurses and 1 social worker) of the center to administer mental health measures (including the Shame Questionnaire) with sexually abused youth. Together, each item on all measures was read aloud, methods for administration were discussed, and the trainer role-modeled administration. All staff then conducted role plays with each other to further practice administration, with coaching and feedback from the trainers. Since the staff believed that most children would not be strong readers, administration included reading each item to the child with the paper in front of them, and then having the child point to a visual with the response options. It was agreed that if there was a strong reader who preferred to read the items, this was allowed. The staff spoke both English as well as other local languages of Zambia (e.g., Nyanja, Bemba, Tonga). Although both boys and girls were brought to the One Stop Center, very few boys completed the Shame Questionnaire. As such, only girls were included in the current record review.

### Measures

#### Selection of measures

Measures were selected for use based on the qualitative study referenced above [39], and on feedback from local stakeholders in Zambia following principles of community-based participatory research [41]. A number of standard instruments were reviewed that closely matched signs and symptoms of the problems described by participants in the qualitative study, and then brought to our Zambian partners for review. Many of the symptoms found in the qualitative study were similar to emotional, interpersonal and behavioral problems seen in sexually abused children in Western settings. In addition to the choice of a trauma measure, the PTSD-RI [30,38], the team believed other problems should also be investigated. Shame was a common response within the qualitative study and local stakeholders believed that shame was an important cultural factor that should be investigated, particularly with the HIV prevalence in Zambia.

#### The Shame Questionnaire

The Shame Questionnaire [37] is an 8-item self-report measure used to assess a child’s feelings of shame in response to sexual abuse. Although there are no known validation studies of the Shame Questionnaire, the original measure has previously been used among children [13,41] and adolescents [13,37,42], who have been sexually abused. It has been used in a randomized controlled trial [41] and longitudinal studies[37,42] among youth in the United States and has demonstrated adequate reliability with an alpha coefficient above 0.85 [37,42]. It was specifically chosen by Zambians who felt that the items appropriately represented the local cultural concept of shame in response to sexual abuse. For the Shame Questionnaire, children were asked to answer how true 8 items were related to the recent abuse using a 3-point Likert scale. Each response was given a different point value, with 0 “not true”, 1 “somewhat true,” and 2 “very true.” A total sum score was generated by summing the responses for the 8 items. Scores could range from 0 to 16.

#### The Post-Traumatic Stress Disorder-Reaction Index

The Post-Traumatic Stress Disorder-Reaction Index (PTSD-RI; [38]), is a self-report measure used to assess traumatic events, experiences and memories of the traumatic event, and post-traumatic stress disorder symptoms related to the event. The PTSD-RI has been used in international settings [43–45] where it has demonstrated adequate internal consistency, concurrent validity and discriminant validity [46–48].

For the current study two sections of the PTSD-RI were used: 1) assessment of 12 different types of traumatic events and 2) assessment of 20 post-traumatic stress disorder symptoms. In addition, there were 18 local mental health outcomes added to the instrument based on findings from a previously conducted qualitative study [39]. As a part of the current study, the PTSD-RI was validated among Zambian children and demonstrated adequate reliability (with Cronbach alpha scores above .90 on all sections), discriminant and concurrent validity [30]. For the 38 symptoms, participants were asked to rate the frequency they experienced the symptom in the previous month using a 5-point scale (0 = none of the time, 1 = little, 2 = some of the time, 3 = much of the time, and 4 = most of the time).

### Procedure

#### Methods for adaptation

Items in the Shame Questionnaire were compared against signs and symptoms mentioned in the qualitative study. Following the methodology of Bolton [49,50], signs and symptoms not in the original Shame Questionnaire but identified by participants in the qualitative study would be added to the measure. The results of the qualitative study, however, indicated that no new items needed to be added. All of the original items from the Shame Questionnaire were also retained. This allowed us to make sure an item originally not mentioned in the qualitative study was actually relevant during the validation study.

#### Translation

The Shame Questionnaire was translated into Nyanja (the most commonly spoken local language), using the following three steps: 1) translation-back translation, 2) group translation, and 3) comparing the initial results to the local vocabulary recorded in the qualitative study. Two translators from the University of Zambia with a background in mental health and an additional multi-lingual translator completed the initial translation-back translation. The translated assessment instruments and validity questions were reviewed item by item by a group of eight local multi-lingual community members. Each item was checked for conceptual understanding. In addition, each item was reviewed for the ability of a child and/or community member with limited education to understand the language with agreement in the final translation. Then, the words chosen by the translators were compared to the words that were a part of the initial qualitative study. If there was a discrepancy between the qualitative and translation terms, the term from the qualitative study was chosen. Although this rarely occurred, it was usually related to making some words more informal.

#### Piloting and implementing of measures

The adapted Shame Questionnaire was piloted for use in the UTH One-Stop Centre starting in August 2007. Piloting was conducted to make sure that translations were understood by a child and/or community members who spoke the language but may have limited education, to allow for the assessors (study interviewers) to practice, and to identify any problems with the implementation process that could then be resolved before formally introducing the questionnaire in the clinic. Each interviewer conducted at least one interview, with some completing two. Results from the pilot indicated no need for substantial changes to the measures and thus the complete measure was implemented as part of standard procedures within the UTH One-Stop Centre.

From September 2007 to January 2008, de-identified data were transferred to the Boston University Center for International Development office in Lusaka once a week by research staff on Teleforms. The Teleforms had a random number ID not linked to their hospital or clinic ID number. The measures were returned to the One Stop Centre the following week. A log book documented which assessment forms were taken away, when they were taken, and when they were returned. This procedure is typical in developing countries where not every separate clinic or office has electronic resources. At the Boston University office, all electronic records were kept on a server with access limited only to members of the research team. In early Spring 2008, the data for this validity study were obtained through a record review of the clinic data.

Institutional Review Board approval for this analysis was obtained from Johns Hopkins University, Boston University, and the University of Zambia Biomedical Research Ethics Committee in Zambia. No consenting process was conducted because the mental health measures were being integrated into normal service at the One Stop Centre and the study was a review of clinic data. This protocol would require a Waiver of Informed Consent, but there was no consent process as it was a medical record review and subjects did not have contact with the researchers. The investigators received de-identified data and could not link this data to any one individual.

### Data Analysis

#### Internal consistency reliability

Cronbach’s alpha scores were used to assess internal consistency reliability of the Shame Questionnaire. A value between .70 and .79 is considered fair, a value between .80 and .89 considered good, and a value .90 and above considered excellent [51,52].

#### Criterion validity

An alternative method of exploring criterion validity based on classical test theory was used for this study as mental health professionals were not readily available in the One Stop Center to conduct professional clinical assessments. In addition, the construct of shame is more of a cluster of symptoms rather than a formal diagnosis with an established gold standard. Criterion validity is establishing a statistical relationship with a measure that is external, but related to the measure itself [52]. In the current study, criterion validity was assessed in three ways. First, we compared the mean scores of the Shame Questionnaire across categorized number of traumatic events experienced. Participants were originally asked on the PTSD-RI to indicate if they experienced a number of different traumatic events. For this study, the number of traumatic events endorsed was totaled into a sum score. As the range of trauma events experienced was positively skewed, the variable was collapsed into a categorical variable with 0 to 1 events experienced indicating 1, 2 events categorized as 2, 3 events as 3, and 4 and above categorized as 4. We combined 0 to 1 events into one category because all participants in the study experienced at least one traumatic event by nature of being at the One Stop Centre. A one-way ANOVA was conducted with the hypothesis that girls with more traumatic experiences would have higher scores on the Shame Questionnaire. Second, trauma types from the PTSD-RI were combined into three categories (witnessing physical abuse, experiencing physical abuse, and experiencing sexual abuse) to examine differences in mean Shame Questionnaire scores between groups. A portion of the sample (almost 40%) endorsed that they did not experience sexual abuse, although the entire sample was comprised of girls who were sexually abused. As such, we hypothesized that girls who endorsed experiencing sexual abuse would have lower levels of shame compared to girls who did not.

We also hypothesized that there would not be a significant difference in Shame Questionnaire scores between girls who endorsed either experiencing or witnessing physical abuse and girls who did not. Finally, correlation analyses were conducted to determine the relationship between the Shame Questionnaire and the PTSD-RI symptom scores, hypothesizing that the Shame Questionnaire would be positively correlated with the PTSD-RI.

#### Construct validity

Construct validity, or the degree to which the measure reflects the underlying latent variable, was determined using IRT analyses. In order to determine the IRT assumption of unidimensionality, an exploratory factor analysis (EFA), based on classical test theory was conducted. Based on the EFA results, using Acer ConQuest 3 [53], a multidimensional 2 parameter rating scale model (RSM) was assessed to determine model fit. The underlying structure of shame was determined through item location (difficulty parameters) from the IRT analyses. Mean Squared Error (MNSQ) infit statistics, which measures the deviance of the empirically observed response probabilities from the model implied expected probabilities were also examined. Reasonable MNSQ fit statistics for RSMs should be above .6 and below 1.4 [54].

#### Sub-analyses

Construct validity was compared between girls who endorsed sexual abuse and girls who did not, using IRT analyses. Similar to the analyses noted above, results from an EFA were used to determine dimensionality for both groups. Based on the EFA results, a multidimensional 2 parameter RSM was assessed to determine model fit for the group of girls who endorsed experiencing sexual abuse. For the sub-sample comprised of girls who did not endorse sexual abuse, a unidimensional 2 parameter RSM was assessed to determine model fit. For both groups, construct validity was determined through item location (difficulty parameters) from the IRT analyses.

## Results

### Demographics

At the time of the record review for this analysis, 690 child intakes had been conducted at UTH. Out of the 690 child intakes, 332 had completed Shame Questionnaire data. Due to the low number of boys with a complete Shame Questionnaire measure (n = 7), the 325 girls with complete Shame Questionnaire data were included in the analysis for this study. Two participants had one item missing from the Shame Questionnaire. This was deemed to be inconsequential in terms of impacting the results of the analysis. Table 1 indicates the demographics of the girls included in this validation record review study (n = 325). Girls in the sample of the record review ranged in age from 6 to 15 years old (M = 12.8). Eighty four percent (n = 273) of the sample were currently enrolled in school. In addition, over 72% of the sample indicated that the current incident of sexual abuse was the first incident that they experienced, with over 45% stating that the incident occurred within the last 72 hours (see Table 1).

**Table 1:** Female Child Participant Demographics and Scale Scores (n = 325).

Age in years, Mean (range)	12.8 (6–15)
Currently in school N (%)	
Yes	273 (84.3%)
No	51 (15.7%)
Sexual Abuse Questions N (%)	
First time it happened	235 (72.3%)
When the most recent abuse occurred	
Within 72 hours	148 (45.5%)
Within the last week	73 (22.5%)
Within the last month	48 (14.8%)
More than a month has passed	42 (13.0%)
Shame mean (standard deviation)	4.87 (4.35)
Trauma types experienced	
Being in a big earthquake	0 (0%)
Being in another kind of disaster	6 (1.8%)
Being in a bad accident	4 (1.2%)
Being in a place where war was happening	0 (0%)
Being hit punched or kicked-**P**	49 (15.1%)
Seeing a family member being hit punched or kicked-**W**	57 (17.5%)
Being beaten, shot at or threatened-**P**	122 (37.5%)
Seeing someone in your community being beaten shot or threatened-**W**	87 (26.8%)
Seeing a dead body in your community-**W**	90 (27.7%)
Having an adult or someone older than you touch you inappropriately-**S**	200 (61.5%)
Hearing about the violent death of someone you know-**W**	28 (8.6%)
Having painful or scary medical procedures	23 (7.1%)

Variable codes for trauma event types: Experiencing physical assault or violence-P; Experiencing sexual assault or violence-S; Witnessing physical assault or violence- W.

### Trauma Event Types

The most frequently endorsed trauma type was being sexually touched (61.5%). Around one-third of the girls reported that they been shot at, beaten or threatened (37.5%) or seen a dead body (27.7%). The mean number of trauma event types experienced was 2.11 (SD = 1.39) with a number of reported trauma types ranging from 0–9 (see Table 1).

### Questionnaire Item Descriptives

Description of all of the items of the Shame Questionnaire with their means and standard deviations are listed in Table 2. The highest endorsed item was *Item 1*, (M = 1.10, SD = .87) and the lowest endorsed item was *Item 6*, (M = .34, SD = .64), suggesting a good range of item distribution of the Shame Questionnaire.

**Table 2:** The Shame Questionnaire Items and Exploratory Factor Analysis Varimax Rotated Factor Matrix of Shame Questionnaire.

Item	Mean	Standard Deviation	Factor 1 Loadings- *Active Outcomes of Shame*	Factor 2 Loadings- *Experience of Shame*
Item 1: I feel ashamed because I think that people can tell from looking at me what happened (tell by look)	1.10	.87	.19(.15,.**70**)	**.66 (.68)**
Item 2:When I think about what happened, I want to go away by myself and hide (away and hide)	.45	.72	.**60(.70, .68)**	.35 (.23)
Item 3: I am ashamed because I feel I am the only person in my school/work who this has happened to (only one happened)	.40	.70	.41(.**42**,. **71**)	**.42** (.19)
Item 4: What happened to me makes me feel dirty (feel dirty)	.83	.85	.23(.15,. **78**)	**.68 (.68)**
Item 5: When I think about what happened, I feel like covering my body.(covering up)	.37	.66	**.76(.87, 76)**	.31(.15)
Item 6: When I think about what happened, I wish I were invisible (wish invisible)	.34	.64	.**92(.87, 77)**	.22 (.11)
Item 7: When I think about what happened, I feel disgusted with myself (disgusted with self)	.70	.82	.27(.14, **80**)	.**69 (.71)**
Item 8: When I think about what happened, I feel exposed. (feel exposed)	.70	.81	.39(.**41**,.**80**)	**.63 (.51)**

Bold indicates factor loading > .40. Parentheses indicates sub-analyses of factor loadings for girls who endorsed sexual abuse, factor loadings for girls who did not endorse sexual abuse.

*Factor loadings for girls who did not endorse sexual abuse are only listed in factor 1.

### Reliability of the Shame Questionnaire

High internal validity was determined for the Shame Questionnaire, with Cronbach’s alpha equal to .87. In addition, there was no improvement in Cronbach’s alpha score for the scale with the removal of any individual item.

### Criterion Validity

Criterion validity for the Shame Questionnaire was measured by comparing the total Shame Questionnaire score across each category of the total number of trauma events experienced. The overall model was significant (F _(3, 324)_ = 6.70, p<.0001) (see Table 3), indicating that Shame Questionnaire scores differ across the number of trauma event types experienced. Bonferroni’s post hoc analysis indicated a significant difference in Shame Questionnaire mean scores between 0–1 and 2 traumatic event types (*p* = .004), 0–1 and 3 traumatic events (*p* = .004) and 0–1 and 4 or more (*p* = .016) traumatic event types, with more traumatic event types experienced resulting in higher mean Shame Questionnaire scores in all cases (see Table 3). All other mean differences were not significant (*p*>.05). These results indicate that more than one traumatic event type experienced is on average more likely to result in a higher score on the Shame Scale compared to those who experience none or one event, suggesting criterion validity of the measure. However, there was no difference on average on the Shame Questionnaire among participants who have experienced more than one traumatic event type.

**Table 3:** Evaluation of Criterion Validity: Analysis of Variance Comparing the Shame Questionnaire Score Between Number of Traumatic Event Types.

	Total Number of Different Types of trauma events, categorized				
	0–1 (n = 133)	2 (n = 87)	3 (n = 59)	4+ (n = 46)	
Shame Questionnaire Scale, mean (SD)	3.61 (4.02)	5.61 (4.40)	5.90 (4.61)	5.80 (4.09)	
Trauma Event Type Comparison	0–1 vs 2	0–1 vs 3	0–1 vs 4+		
Bonferroni post hoc significant mean difference (standard deviation)	M = -2.00 (.58), *p* = .004	M = -2.29 (.66), *p* = .004	M = -2.20 (.73), *p* = .016		
	Sum of Squares	Df	Mean Square	F	p-value
Between groups	361.56	3	120.52	6.70	<.0001
Within groups	5775.01	321	18.00		
Total	6136.57	324			

To further explore criterion validity, different trauma types were compared with the Shame Questionnaire score through independent samples t-tests. Trauma type variables were created from the trauma events of the PTSD-RI (see Table 1). Each variable was dichotomized. The t-test indicated a significant difference with experiencing any type of physical abuse, with a mean Shame Questionnaire score of those without a physical abuse history (M = 3.98, SD = 4.14) 1.90 points lower than girls with a history of physical abuse (M = 5.88, SD = 4.38) (t_(323)_ = -4.02, p<.0001; 95% CI -2.82,-.97). Results revealed a significant difference between girls who witnessed physical assault or abuse, with a mean Shame Questionnaire score of those without a history of witnessing physical abuse (M = 4.24, SD = 3.96) 1.26 points lower than girls with a history of witnessing physical abuse (M = 5.50, SD = 4.63) (t_(323)_ = -2.63, p<.0001; 95% CI -2.19,-.32). There was not a significant difference in mean Shame Questionnaire scores between girls who endorsed experiences of sexual abuse (M = 4.62, SD = 3.59) and those who did not endorse such experiences (M = 5.27, SD = 5.34) (p>.05).

Results found a significant positive relationship (p<.0001) between the Shame Questionnaire and PTSD-RI, with *r* = .41 indicating that higher Shame Questionnaire scores are associated with higher scores on the PTSD-RI.

### Construct Validity

#### Unidimensionality assumption

A simple factor solution with two initial factors was obtained using Varimax rotation and a factor loading cutoff of .4. Kaiser Myer Olkin (KMO) measure of sampling adequacy was superb with a score of .85 [55] and a significant Bartlett’s test of sphericity (χ^2^_(28)_ = 1158.31, p<.05) suggesting a factor analysis was appropriate to conduct with the data. Two factors were suggested from initial Eigenvalues over 1. The first factor indicated 52.32% of the variance and the second factor indicated an additional 13.36% of the variance. Factor one had four items with loadings above .4 (*Items 2*, *3*, *5*, *and 6*). Factor two had five items (*Items 1*, *3*, *4*, *7*, *and 8*) (Table 2). *Item 3* had factor loadings above .4 in both factor 1 and factor 2. The higher factor loading (Factor 2) was used to determine fit for *Item 3*. Results of the EFA were suggestive of multidimensionality and made sense conceptually with a factor related to an *experience of shame (Factor 2)* and a factor related to *active outcomes of shame (Factor 1)*.

#### Multidimensional IRT RSM

Using Acer Conquest 3 [53], a multidimensional RSM was estimated to examine individual item properties and examine the dimensional structure of shame with the data. Overall, MNSQ fit statistics suggested overall good fit of the model. However, one item (*Item 6*) had a MNSQ estimate (MNSQ = .58) that was slightly outside of the acceptable range, indicating that the individual response of this item does not fit well with the Shame Questionnaire measurement model. In addition, *Item 1* and *Item 6* had the lowest and highest difficulty parameters in the model, (-1.10, 1.18), respectively (see Table 4).

**Table 4:** Multidimensional Rating Scale Model with Unstandardized Parameters.

Item	Difficulty Parameter	MNSQ* Infit Statistic
Factor 1: (Active Outcome of Shame)		
Away and Hide	-.33	.91
Covering up	.10	.63
Wish invisible	.24	.58
Factor 2: (Experience of Shame)		
Tell By Look	-1.10	1.15
Only One Happened	1.18	1.20
Feel Dirty	-.32	.94
Disgusted with self	-.13	.91
Feel exposed	.11	.85

*Mean Squared Error.

#### Sub-analyses for girls who endorsed sexual abuse

For girls who endorsed experiencing sexual abuse, a simple factor solution with two initial factors was obtained using Varimax rotation and a factor loading cutoff of .4. The Kaiser Myer Olkin (KMO) measure of sampling adequacy was superb with a score of .80 [55] and a significant Bartlett’s test of sphericity (χ^2^_(28)_ = 610.97, p<.05) suggesting a factor analysis was appropriate to conduct with the data. Two factors were suggested from initial Eigenvalues over 1, with the first factor indicating 44.33% of the variance and the second factor 19.04% of the variance. Factor 1 had 4 items with loadings above .4 (*Items 2*, *3*, *5 and 6*). Factor 2 had 4 items with loadings above .4 (*Items 1*, *4*, *7 and 8*) (Table 2). *Item 8* had factor loadings above .4 in both factor 1 and factor 2. The higher factor loading (Factor 2) was used to determine fit for *Item 8*. Results of the EFA were suggestive of multidimensionality and were similar to the two factors of the whole sample, with the exception of *Item 3*.

Using Acer Conquest 3 [53], a multidimensional RSM was estimated to examine individual item properties and examine the dimensional structure of the Shame Questionnaire among girls who endorsed experiencing sexual abuse. MNSQ fit statistics suggested overall good fit of the model. In addition, *Item 4* and *Item 6* had the lowest and highest difficulty parameters in the model, (-.55, 1.55) respectively (Table 5).

**Table 5:** Sub-Analysis: Multidimensional Rating Scale Model with Unstandardized Parameters for Girls who Endorsed Sexual Abuse vs. Unidimensional Rating Scale Model with Unstandardized parameters for Girls who Did Not Endorse Sexual Abuse.

Item	Difficulty Parameter endorsed sexual abuse vs not endorsed	MNSQ Infit Statistic for endorsed sexual abuse vs not endorsed
Factor 1		
Away and hide	-.31, .39	1.02, .97
Only one happened	1.03, .26	1.07, .83
Cover up	.21, .63	.61, .59
Wish invisible	1.55, .79	.80, .50
Factor 2		
Tell by look	-.13, -1.19	1.09, 1.42
Feel dirty	-.55,-.31	1.12, 1.00
Disgusted with self	.11,-.25	.89, 1.04
Feel exposed	.22,-.50	.89, .86

#### Sub-analyses for girls who did not endorse sexual abuse

For girls who did not endorse experiencing sexual abuse a simple factor solution with one initial factor was obtained using Varimax rotation and a factor loading cutoff of .4. The Kaiser Myer Olkin (KMO) measure of sampling adequacy was superb with a score of .86 [55] and a significant Bartlett’s test of sphericity (χ^2^_(28)_ = 610.39, p<.05) suggesting a factor analysis was appropriate to conduct with the data. One factor was suggested with initial Eigenvalues over 1, indicating 61.64% of the variance (Table 4).

Using Acer Conquest 3 [53], a unidimensional RSM was estimated to examine individual item properties and examine the dimensional structure of the Shame Questionnaire with the data. Overall, MNSQ fit statistics suggested overall good fit of the model. However, two items (*Item 5* and *Item 6*) had a MNSQ estimate (MNSQ = .59 and MNSQ = .50) that was outside of the acceptable range, indicating that the individual response of these items do not fit well with the Shame Questionnaire measurement model among girls who did not endorse sexual abuse. In addition, *Item 1* and *Item 6* had the lowest and highest difficulty parameters in the model, (-1.19, .79), respectively (Table 5).

## Discussion

This record review study aimed to test the validity and reliability of the Shame Questionnaire among traumatized girls in Zambia. Searches of the English literature indicated that no previous study has validated a measure of shame in a non-Western low and middle income context. Results of our study demonstrate that the Shame Questionnaire is a reliable measure with adequate criterion and construct validity.

Among the whole sample, the EFA suggests a 2-factor structure of the Shame Questionnaire among Zambian girls who have experienced sexual abuse. Conceptually, the results seem to indicate a factor for the *experience of shame* and a factor for the *active outcomes of shame*. The results from the multidimensional RSM suggest that *Item 6* (When I think about what happened, I wish I were invisible) was slightly outside of the acceptable range for adequate fit. The measurement error of this item could be due to a number of potential factors. Although rigorous methods were taken to ensure that the conceptual meaning was maintained in translation, it remains possible that participants did not understand the meaning of the question or that the item was not relevant for this population. Additionally, similar to the western notion of “*wishing one were invisible*,” in Zambia there may be multiple ways of understanding the meaning of this item. In addition, this item seems to be less concrete than the rest of the items of the Shame Questionnaire. Perhaps, Zambian girls have less of a frame of reference for understanding the concept given the daily demands of life in LMIC. Future research among this population should examine other potential reasons for lack of fit for this item.

Findings from the item response theory analysis for the whole sample indicated a wide distribution of item location of the Shame Questionnaire, with both low and high difficulty parameters in each of the two factors. These results suggest that the measure is an appropriate tool to assess a range of shame severity among Zambian girls. However, the difficulty parameters of the two items with the lowest and highest amounts of the Shame Questionnaire seem to be substantially outside the range of the other items, which were between-.33 and .24. *Item 1* (I feel ashamed because I think that people can tell from looking at me what happened) had the lowest difficulty parameter indicating that girls with very little shame endorsed the item. Conversely, *Item 3* (I am ashamed because I feel I am the only person in my school/work who this has happened to) had the highest difficulty parameter, indicating only girls with very high amounts of shame endorsed this item. Because sexual abuse and HIV is so pervasive in Zambia there may be very few girls who believe that they are the only ones who have had this experience. Thus, only those with extreme levels of shame actually believe that they are in fact the only ones who have experienced sexual abuse. At the same time, girls in Zambia may receive messages that once they are sexually abused people can tell and could potentially account for the finding that one does not need that much shame to believe this. Future research should use item response theory methods among this population to understand the relevance of these items among Zambian girls who have been sexually abused.

Zambian youth in the current record review study had a mean total score of 4.87 on the Shame Questionnaire, which is considered in the high range and above the mean cutoff of 3 compared to United States youth [37]. A study of US male and female youth (over 75% of the sample was comprised of females) indicated that a total score above 3 is high indicating 3 out of 4 items were endorsed with moderate amounts of shame [37]. In the current record review, Zambian girls who have experienced sexual abuse are likely to experience extremely high amounts of shame as an outcome of trauma. In addition, results indicated that more than one traumatic event resulted in higher amounts of shame, although there was no difference in amount of shame between 2 or more traumatic events. This suggests that shame should be a target of treatment for girls with more than one traumatic experience, regardless of the number of events experienced above one event. In addition, a longitudinal study among US youth demonstrated that higher Shame Questionnaire scores predicted an increase of intrusive memories which, in turn, is associated with challenges in processing the traumatic event [37]. As such, this also gives support for shame as a target for intervention efforts among youth in Zambia who have experienced trauma.

Although results from the current record review provided support for criterion validity in terms of number of trauma event types experienced, the findings were mixed for criterion validity by trauma type. Girls who did not endorse experiencing sexual abuse did not have significantly higher mean Shame Questionnaire scores than girls who did report sexual abuse. This finding may be due to underreporting of sexual abuse, a common challenge [56,57]. Our record review indicated that many participants reported that they had not been sexually abused even though 100% of the sample had intakes at the center due to previous sexual abuse. In order to even be seen at the center, previous steps were taken which further confirmed sexual abuse, such as someone reporting the incident to a victim support unit and/or registering the female child at the University Teaching Hospital shortly after the incident. However, the high number of girls who did not endorse being sexually abused could be due to a local phenomenon of girls having a “sugar daddy” or someone much older who sexually abuses them. The abuse is often paired with buying the girl minor things (such as food), and/or paying school fees making the perception of the relationship as the older man “taking care of her” understandable.

Both the factor structure and item location was slightly different between girls who did and did not endorse sexual abuse. However, there was not a significant difference in mean Shame Questionnaire scores among girls who did not endorse sexual abuse compared to girls who did, although those who did not endorse sexual abuse had slightly higher mean Shame Questionnaire scores. The difference in mean Shame Questionnaire scores makes sense knowing that the whole sample experienced sexual abuse as those who denied the abuse may have more shame and be less likely to disclose the experience. However, the lack of significance but differences in factor structure and item location suggests that there are other factors that may be contributing to why some girls would report abuse and others would not. Similar to western settings, girls who are sexually abused by a family member or someone they know may be less likely to endorse the experience due to potential blame from the family and resources provided by the perpetrator [58]. On the other hand, girls who are abused by a stranger may be more likely to disclose the experience because the family is more supportive and responsive. It is also interesting that the fit of *Item 6* (When I think about what happened, I wish I were invisible) was within adequate range for girls who reported sexual abuse but outside of adequate range for girls who did not report sexual abuse. This suggests measurement error in this item specifically among this sub-sample and indicates that this item may not be a reliable indication of the concept of shame among girls who do not report sexual abuse but have been sexually abused. Perhaps *wishing one were invisible* is a common symptom for girls who see the act as abuse; whereas those who perceive the abuse as a relationship with a “boyfriend” or “sugar daddy” would not wish for this to be invisible deeming this item not relevant for this sub-sample. Results indicating differences from the sub-analyses suggest that there may be clinical implications in terms of treatment of shame symptoms for the two groups.

## Limitations and Conclusions

Although this study has a number of strengths, there are a few limitations that should be mentioned. The sample of the current study is comprised only of female children. Future research should examine the role of shame and validate the Shame Questionnaire for male children in Zambia, especially given previous research has suggested that prevalence of child sexual abuse among males in sub-Saharan Africa may be high [59]. In addition, while the majority of the sample experienced multiple traumatic events, all youth in the record review were sexual abuse survivors limiting the generalizability. Finally, as almost 40% of the sample did not reliably report their sexual abuse history, findings from the current study are limited as they may indicate that they also did not reliably endorse other trauma experiences or symptoms of shame. For Zambian health professionals, this suggests the need for more objective or collateral sources to confirm sexual abuse. However, this is a common challenge within sexual abuse reporting as there are often individuals who do not “see” the sexual abuse as abuse [22,59].

In conclusion, this record review validation study suggests the general utility of the Shame Questionnaire among Zambian girls and demonstrates the need for more psychometric studies in Zambia and other culturally similar sub-Saharan Africa countries. IRT as a research method in this study allowed for the exploration of individual items and gave a different perspective of the construct validity of the Shame Questionnaire among Zambian girls. As such, results from this study suggest that both CTT and IRT can be complementary approaches to validate instruments in LMIC. Further, As the “sugar daddy” phenomenon has been noted in other sub-Saharan-African countries (e.g. Tanzania and South Africa), [22,60], future research should examine the type of the relationship between the female child and abuser and the relevance in terms of symptoms of shame. The overall results suggest that shame is an important cultural construct in Zambia and potentially other countries in sub-Saharan Africa, especially in countries with high HIV prevalence based on data showing a relationship between shame and HIV risk behavior [16,19,20].

## Supporting Information

S1 Data (ZIP)
